# Electric Light Notes

**Published:** 1881-07

**Authors:** 


### ELECTRIC LIGHT NOTES.

It is reported that the Post-office department in Paris contemplates introducing the electric light in place of gas.

The New York Board of Fire Insurance Underwriters have decided to rate as "specially hazardous" all buildings using the electric light, unless the wires are insulated.

Mr. W. H. Preece suggests that the flow of tidal waters, and in elevated stations wind power, might be used to reduce the cost of producing the current for the electric light.

An effort is being made by the Police Committee of Philadelphia to light Chesnut street by electricity at a cost of $5,000 per annum. The *Ledger* figures that at this rate it would cost a million and a quarter dollars to light the city.

According to the *Gas Engineer*, the principal streets of Newcastle, Eng., are being lighted by the "Swan" incandescent lamp. The District Gas Company, however, accepting the challenge, have erected one of Bray's large gas lamps.

Akron, Ohio, has been experimenting with electric lights placed on high towers as a means of lighting the town. The experiment has proved so successful that five more towers are to be erected. It is also reported that a similar plan is to be adopted in Cleveland.

The Gas Commission have awarded a contract to the Brush Electrict Light Co., to light the following streets for one year for the sum of $7,400—14th street, from 4th to 5th avenue, 5th avenue from 14th to 34th streets ; 34th street, from 5th avenue to Broadway, and Broadway from 14th tô 34th streets ; also two large lights in Union and Madison Squares. It is also expected that these lights will be in operation about July 1st.

Mr. H., Joel, of London, has invented an electric lamp which holds a position midway between the arc and the incandescent lights. In principle it is the same as that of Werdermann, and consists of a pencil of carbon pressing against a copper terminal, the carbon being heated by a current of electricity. The lamp can be placed in a vacuum globe although this is not necessary to the development of good light.

The *Review of Gas and Water Engineering* states that according to Mr. Preece, the cost of working the sixteen electric lamps in the South Kensington Museum from June 22 to December 31, a total of 359 hours, was about $334, being at the rate of 93 cents per hour of light. The cost of gas would have been $3.87 per hour. This, however, does not include interest, wear and tear, etc., but deducting these, he shows a saving of something over $1,500 per annum in favor of electricity.

The Paris Exhibition of Electricity which is to open on the 1st of August is attracting very general attention, and it is expected that there will be a fair representation of American exhibitors. The State Department at Washington has received a letter of information from the Acting U. S. Commissioner-General to the Exhibition, in which we note the following :

Exhibitors are requested to make immediate application to the Commissioner for the space desired, giving a description of their exhibit, for which official labels will be furnished. All goods for exhibition will be carried by the French railways at full rates to Paris, but will be taken back to the port of reshipment gratis. Motive power will be supplied at a moderate cost, but each exhibitor must furnish his own electrical currents. Exhibits will be received on and after July 1.

On May 3 that district of London lighted by the Brush electric lamps, from Blackfriars to the middle of Cheapside, was left in darkness for several hours. The trouble seems to have been a defect in the insulation of the wires, which was not apparent until the occasion of a heavy rainstorm. Anti-electric light Journals are making considerable of this accident. The *Gas Engineer*, however, rather favors the Brush system, both on account of quality of the light and the arrangement of the lamps.

